# Utilization of a Strongly Inducible *DDI2* Promoter to Control Gene Expression in *Saccharomyces cerevisiae*

**DOI:** 10.3389/fmicb.2018.02736

**Published:** 2018-11-16

**Authors:** Aiyang Lin, Chuanwen Zeng, Qian Wang, Wenqing Zhang, Mengyao Li, Michelle Hanna, Wei Xiao

**Affiliations:** ^1^Beijing Key Laboratory of DNA Damage Response and College of Life Sciences, Capital Normal University, Beijing, China; ^2^Department of Biochemistry, Microbiology and Immunology, University of Saskatchewan, Saskatoon, SK, Canada

**Keywords:** *Saccharomyces cerevisiae*, *DDI2*, promoter, transcriptional regulation, cyanamide

## Abstract

Regulating target gene expression is a common method in yeast research. In *Saccharomyces cerevisiae*, there are several widely used regulated expression systems, such as the *GAL* and Tet-off systems. However, all current expression systems possess some intrinsic deficiencies. We have previously reported that the *DDI2* gene can be induced to very high levels upon cyanamide or methyl methanesulfonate treatment. Here we report the construction of gene expression systems based on the *DDI2* promoter in both single- and multi-copy plasmids. Using *GFP* as a reporter gene, it was demonstrated that the target gene expression could be increased by up to 2,000-fold at the transcriptional level by utilizing the above systems. In addition, a *DDI2*-based construct was created for promoter shuffling in the budding yeast genome to control endogenous gene expression. Overall, this study offers a set of convenient and highly efficient experimental tools to control target gene expression in budding yeast.

## Introduction

Budding yeast *Saccharomyces cerevisiae* is a prototype model lower eukaryotic organism widely used to study genomics and gene expression (Botstein and Fink, 2011). Several regulatory systems have been employed to experimentally control target gene expression in budding yeast, among which the *GAL* and Tet-off systems are the most popular (Nevoigt, 2008). The galactose induction system in budding yeast serves as a prototypical transcriptional induction system (Lohr et al., 1995; Matsuyama et al., 2011). It is one of the most characterized forms of signal transduction in eukaryotes at different levels (Lohr et al., 1995). The expression of *GAL7* and divergently transcribed *GAL1-GAL10* genes is repressed by glucose, in a non-induced state in raffinose and highly induced when galactose is the sole carbon source (Nehlin et al., 1991; Huibregtse et al., 1993; Lohr et al., 1995). Although it is the most-used regulatory system for inducing foreign gene expression in budding yeast, there are a few obvious limitations. Firstly, since glucose is a strong repressor, it requires that raffinose and galactose in the culture medium be glucose-free, which is rather expensive and impractical for large-scale production. Secondly, yeast cells must be deprived of all glucose present in the medium prior to galactose induction (Mann and Grunstein, 1992; Lenfant et al., 1996), which is time consuming. Thirdly, since galactose is not a preferred carbon source for budding yeast to grow, to achieve an efficient and timely induction of the target gene, an ideal induction scheme includes growing yeast cells in glucose first, followed by shifting to raffinose and then to galactose (Lohr et al., 1995). Finally, certain laboratory budding yeast strains are Gal^−^ and do not support the *GAL* induction system.

The tetracycline regulatory systems (Tet-on and Tet-off), which were initially found from the bacterial transcription factor Tet repressor (TetR) combined with a TetR-responsive promoter (Berens and Hillen, 2003; Schmidt et al., 2014), have been widely applied to regulate gene expression in eukaryotes (Baron and Bujard, 2000). In the Tet-off system the target gene expression is suppressed in the presence of tetracycline (Tet) or its derivative doxycycline (Dox) and can be activated by tTA, a Tet-controlled transactivator protein, binding to a Tet-responsive promoter element (TRE) in the absence of Tet or Dox (Freundlieb et al., 1997; Baron and Bujard, 2000). The Tet-off system has not only been employed to create a genome-wide gene regulatory system to study essential genes in budding yeast, gaining some popularity in studying individual yeast genes (Dingermann et al., 1992; Park and Morschhauser, 2005; Zilio et al., 2012), but has also been frequently used in a variety of different organisms ranging from bacteria to mammals (Gatz et al., 1991; Gossen and Bujard, 1992; Lewandoski, 2001; Bertram and Hillen, 2008; Sheng et al., 2010). However, there are a few issues that limit its application. First, the Tet-off system appears to have a high basal expression level even in the presence of Dox (Baron and Bujard, 2000), and its efficacy in regulating different target genes varies. Secondly, to utilize this system, the tTA gene must be introduced into the host cells first. Thirdly, The Tet-off system is inconvenient for large-scale induction, as Dox is rather expensive.

*DDI2* and *DDI3* are two identical budding yeast genes located on different chromosomes, which were identified through a genome-wide microarray analysis of budding yeast gene expression in response to the DNA-damaging agent methyl methanesulfonate (MMS). They displayed the highest induction (>100-fold) after treatment with a sublethal dose of MMS (Fu et al., 2008), and was named after *DDI1* (Liu and Xiao, 1997). It turns out that *DDI2/3* encodes a cyanamide hydratase (Li et al., 2015). Interestingly, the *DDI2/3* gene is also highly induced after treatment with cyanamide (CY), as measured by a *lacZ* reporter assay (Li et al., 2015).

We thought that the *DDI2* promoter could be developed into a useful experimental tool in controlling endogenous or heterologous gene expression in budding yeast that would be advantageous over the current regulatory systems. Firstly, the *DDI2* gene expression is barely detectable under normal culture conditions and can be highly induced by CY or MMS. Secondly, the induction appears to be rapid and reproducible, and in a linear relationship within a broad range of CY and MMS doses, as judged by the *lacZ* reporter assay (Li et al., 2015). Thirdly, as CY is a simple compound and has been used as crop fertilizer, it is inexpensive and hence can be applied to large-scale production. Finally, the induction process does not require additional regulatory elements and is most likely applicable to all budding yeast strains. In this report we present our construction and characterization of reagents utilizing the *DDI2* promoter to control target gene expression.

## Materials and Methods

### Media and Yeast Strains

Rich yeast extract-peptone-dextrose (YPD) medium was used to culture yeast cells. A synthetic dextrose (SD)-Ura medium was used to select yeast transformants.

The haploid budding yeast strain BY4741 (*MAT*a *his3*Δ1 *leu2*Δ0 *met15*Δ0 *ura3*Δ0) was used in this work. To validate the *DDI2*-based promoter shuffling strategy, an *mCherry-MX6* genetic element was integrated into the *HIS3* locus of BY4741 to form WXY3649 as previously described (Tian et al., 2013).

### Plasmid Construction

The single- and multi-copy plasmids YCpU-P_DDI2_ and YEpU-P_DDI2_ were constructed by first cloning the *ADH1* terminator sequence from plasmid pGAD424 (Fields and Song, 1989) using a pair of oligonucleotides 5′-CCCACTAAGCTTGCGAATTTCTTATGATTT-3′ and 5′-TTATATAAGCTTCCGGTAGAGGTGTGGTCA-3′ as a *Hin*dIII (underlined) fragment into plasmids YCplac33 and YEplac195 (Gietz and Sugino, 1988), respectively. The resulting plasmids were then used to clone an 888-bp *DDI2* promoter region amplified by primers 5′-CCAACTGAATTCTTCAAAGGTTAAACTCGC-3′ and 5′-GGTGGG GAATTCGATTGATTCTTTTGAAGA-3′ from genomic DNA and cleaved by *Eco*RI (underlined) to form YCp-P_DDI2_ and YEp-P_DDI2_. The resulting plasmids were confirmed by restriction analysis and sequencing entire inserts.

In order to validate these two expression vectors, a GFP sequence was amplified by primers 5′-CATAGCGGTACCATGAGTAAAGGAGAAGAA-3′and5′-CCGTGACTGCAGTTATTTGTAGAGCTCATC-3′ from pGFPuv (Clontech) and then cloned into the *Kpn*I and *Pst*I sites within the multiple cloning sites (MCS) to form YCp-P_DDI2_-GFP and YEp-P_DDI2_-GFP.

To construct the *DDI2* promoter-shuffling vector, a *Sal*I-*Eco*RI fragment containing the *URA3* gene was first cloned into pBluescript SK (Stratagene) to form pBS-URA3. A 470-bp *DDI2* promoter sequence was amplified by 5′-CGCCGCGGTACCTTAGACTATGTCTATAAT-3′ and 5′-GAGGCTGTCGACCCCAGCTTGTACTCCGTA-3′ from genomic DNA, cleaved by *Kpn*I and *Sal*I (underlined) and cloned into pBS-URA3 as an upstream *DDI2* promoter element (P_DDI2_U) to form pBS-PDDI2U-URA3. A 557-bp *DDI2* promoter sequence was amplified by 5′-CGCCGCGGATCCGCGATAGTTCCCGAATGT-3′ and 5′-GAGGCTGAGCTCGATTGATTCTTTTGAAGA-3′, cleaved by *Bam*HI and *Sac*I (underlined) and cloned into pBS-PDDI2U-URA3 as a downstream *DDI2* promoter element (P_DDI2_D) to form pDUD (see Figure 6A).

### Yeast Genomic DNA Extraction

A yeast genomic DNA extraction method as described in Hanna and Xiao (2006) was followed with modifications. Briefly, a single colony of yeast cells was picked and suspended in 100 μl of lysis buffer (20 mM NaOH and 0.1 mg/ml RNase A), then incubated at 105°C for 20 min. The sample was then mixed by vortexing, and centrifuged at 12000 *g* for 2 min. The supernatant was used as a template for genomic PCR.

### Yeast Survival Assay

Liquid killing experiments were performed as described (Xu et al., 2014). Briefly, overnight yeast cells were used to inoculate fresh YPD medium at OD_600 nm_ = 0.2–0.3 and incubated at 30°C for 2 h. Cells were then aliquoted into 5-mL samples and treated with MMS or CY at given doses for another 2 h before being collected, washed, diluted, spread onto YPD plates and incubated at 30°C for 3 days. Number of colonies with untreated cells was taken as a reference (100% survival).

### Yeast Transformation

Yeast transformation followed a lithium acetate method (Ito et al., 1983) as described (Gietz and Wood, 2006). Briefly, yeast cells were cultured at 30°C overnight, diluted tenfold into 10 ml of fresh YPD and then cultured for another 4 h. 1.5 ml cells were collected by centrifugation at 3000 *g* for 2 min, washed with water twice and then suspended in 100 μl 0.1 M LiAc and incubated at 30°C for 10 min. Cells were collected again by centrifugation and resuspended in 60 μl water, to which 360 μl 50% PEG, 55 μl 1 M LiAc, 75 μl ssDNA and 200 ng plasmid DNA were added, well mixed, and incubated for 30 min at 30°C and 30 min at 42°C. Finally, cells were collected by centrifugation, washed twice with water and plated on a selective medium.

To integrate the DUD cassette at the *P_HIS3_-mCherry* locus in WXY3649, the DUD cassette was amplified by primers 5′-TCTTGGCCTCCTCTAGTACACTCTATATTTTTTTATGCCTTAGACTATGTCTATAATAT-3′ and 5′-CTTGCTCACCATGGTGGCGACCGGTAGCGCTAGCGGATCGATTGATTCTTTTGAAGAGA-3′ using plasmid pDUD as the template. The resulting PCR product was used to transform WXY3649. The selected individual transformants were screened by genomic PCR and one confirmed strain, WXY3880, was used to select the *P_DDI2_-URA3* pop-out on 5′-fluoroorotic acid (5-FOA) as previously described (Tian et al., 2013).

### Yeast RNA Extraction and Real-Time RT-PCR (qRT-PCR)

Yeast cells cultured in YPD or SD-Ura overnight were subcultured into the same medium at OD_600 nm_ = 0.2∼0.3. After a 2-h incubation, CY or MMS was added and the incubation was continued for another 2 h. Cells were collected by centrifugation and total RNA was extracted using a HiPure Yeast RNA Kit (Geneaid, RBY050) and used to synthesize cDNA using an iScript^TM^ cDNA Synthesis Kit (Bio-Rad). qRT-PCR was performed using iQ^TM^ SYBR Green Supermix (Bio-Rad). Primers used to measure genes of interest along with internal controls are given in the figure legends. The results were analyzed by using the 2^−ΔΔC^_T_ method as described (Livak and Schmittgen, 2001) and expressed as relative change in the target gene expression.

### Yeast Whole-Cell Protein Extraction and Western Blot Analysis

Yeast cells were cultured in 10 ml SD-Ura selective medium overnight and subcultured into 50 ml fresh SD-Ura medium at OD_600 nm_ = 0.2∼0.3. After a 2-h incubation, cells were split into 10-ml cultures to which CY or MMS was added. After another 2-h incubation, cells were collected by centrifugation at 12,000 rpm for 2 min, washed twice in H_2_O. The pellet was resuspended in 1-ml protein lysis buffer (30 mM Tris-HCl pH7.5, 150 mM NaCl, 2 mM MgCl_2_, 0.1% NP40, 1 mM EDTA, 10% glycerol) containing freshly added protease inhibitors (Roche) and 200 μl acidified glass beads. Cells were lysed by placing them into a liquid nitrogen tank for 1 min and then using a bead beater to break the yeast cells (repeating three times each for 5 min). Samples were centrifuged at 12,000 rpm for 2 min and the supernatant was used for SDS-PAGE and western blot analysis.

### Microscopy

Yeast colonies were picked, cultured overnight in SD-Ura and then subcultured to OD_600 nm_ = 0.2∼0.3). After a 2-h incubation, CY or MMS was added to the desired concentration and the incubation was continued for another 2 h. 1.5-ml cells were collected by centrifugation at 3000 *g* for 2 min, washed twice with 1 × PBS and observed by microscopy. Fluorescence was observed under a Zeiss LSM780 confocal microscope and processed by Image J software. For each treatment, cells were counted in at least three independent fields with more than 100 cells per field on average and the results are presented as percentage of cells with fluorescent signals.

## Results

### The *DDI2/3* Gene Is Highly and Rapidly Induced by CY and MMS

**FIGURE 1:**
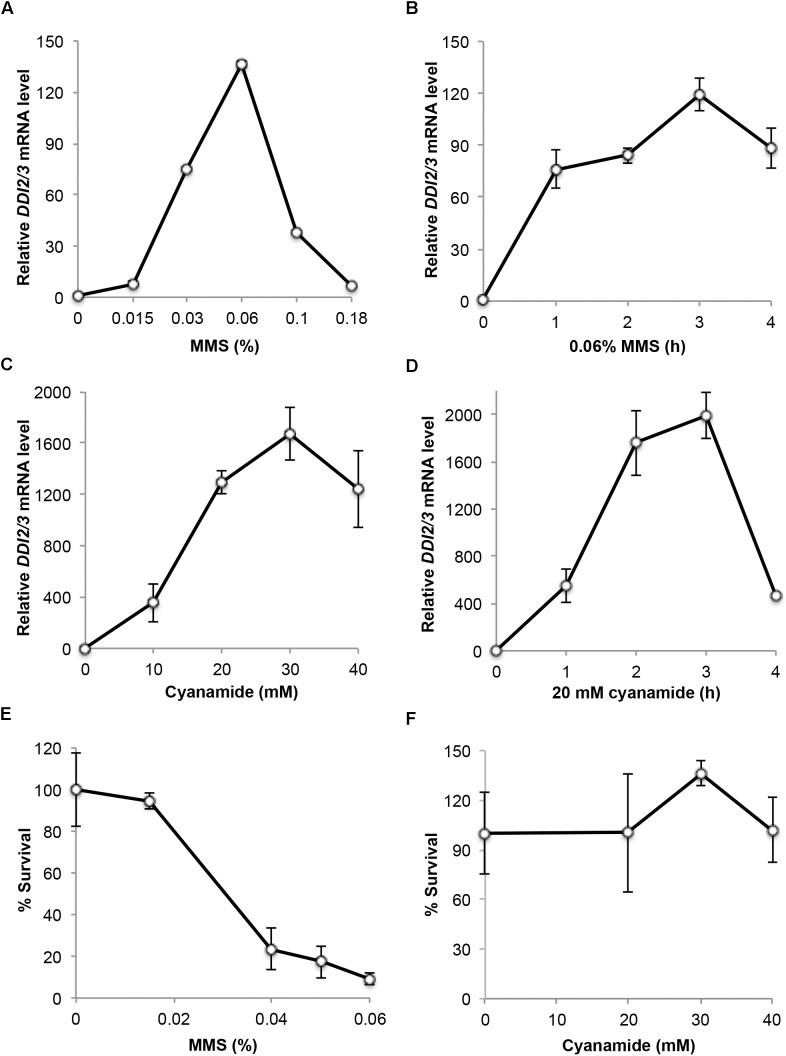
Characterization of endogenous *DDI2/3* gene expression and induction. **(A–D)** Endogenous *DDI2/3* gene expression in BY4741 cells was measured by qRT-PCR with *DDI2/3*-specific primer pairs 5′-GTTGTTCCGCCTCCAAACAGTG-3′ and 5′-CTGCATAGTCCTGATTTCCACC-3′. The yeast *UBC6* mRNA was used as an internal control (Teste et al., 2009). The relative *DDI2/3* mRNA level with untreated cells in each experiment was set as 1. **(A)** Dose response to MMS treatment for 2 h. **(B)** Time course response to 0.06% MMS. **(C)** Dose response to CY treatment for 2 h. **(D)** Time course response to 20 mM CY. **(E)** Cell survival after MMS treatment at the given doses for 2 h. **(F)** Cell survival after CY treatment at the given doses for 2 h. All data are an average of at least three independent experiments with standard deviations shown as error bars.

To assess the dynamics of *DDI2/3* induction after CY or MMS treatment, we performed a real-time RT-PCR (qRT-PCR) assay. As shown in Figure 1, the *DDI2/3* gene can be induced by MMS more than 100-fold, consistent with a previous report (Fu et al., 2008). The optimal MMS dose for induction is at 0.06% (Figure 1A) and 1-h treatment at this dose achieved 75-fold induction (Figure 1B). To our surprise, the *DDI2/3* gene can be induced by cyanamide up to 2000-fold at the transcriptional level, and more than 500-fold induction can be achieved within an hour after 20 mM CY treatment (Figures 1C,D).

MMS is a well-established DNA-damaging agent (Friedberg et al., 2006) that may be toxic to host cells. Indeed it was found that 0.06% MMS treatment for 2 h resulted in approximately 90% cell death, while 0.015% MMS treatment for 2 h has very little if any toxicity (Figure 1E). On the other hand, cyanamide has been used as a herbicide and fungicide due to its mild toxicity (Güthner and Mertschenk, 2000). Little is known about the relative sensitivity of yeast to cyanamide, although we previously demonstrated that budding yeast cells lacking the cyanamide hydratase activity displayed an enhanced sensitivity to cyanamide (Li et al., 2015). Figure 1F indicates that treatment of yeast cells by up to 40 mM CY for 2 h did not cause noticeable cell death. Hence, we conclude that cyanamide has no toxic effect to wild-type cells under optimal *DDI2/3* induction conditions.

### Construction of Gene Expression Vectors Based on the DDI2 Promoter

To construct *DDI2* promoter-based cloning vectors, plasmids YCplac33 (YCp, Amp^*R*^, MCS, *URA3*) and YEplac195 (YEp, Amp^*R*^, MCS, *URA3*) (Gietz and Sugino, 1988) were used as single- and multi-copy plasmid backbones, respectively. These plasmids can be selected by ampicillin resistance in bacterial cells and by uracil prototrophy in yeast *ura3* mutant cells.

**FIGURE 2:**
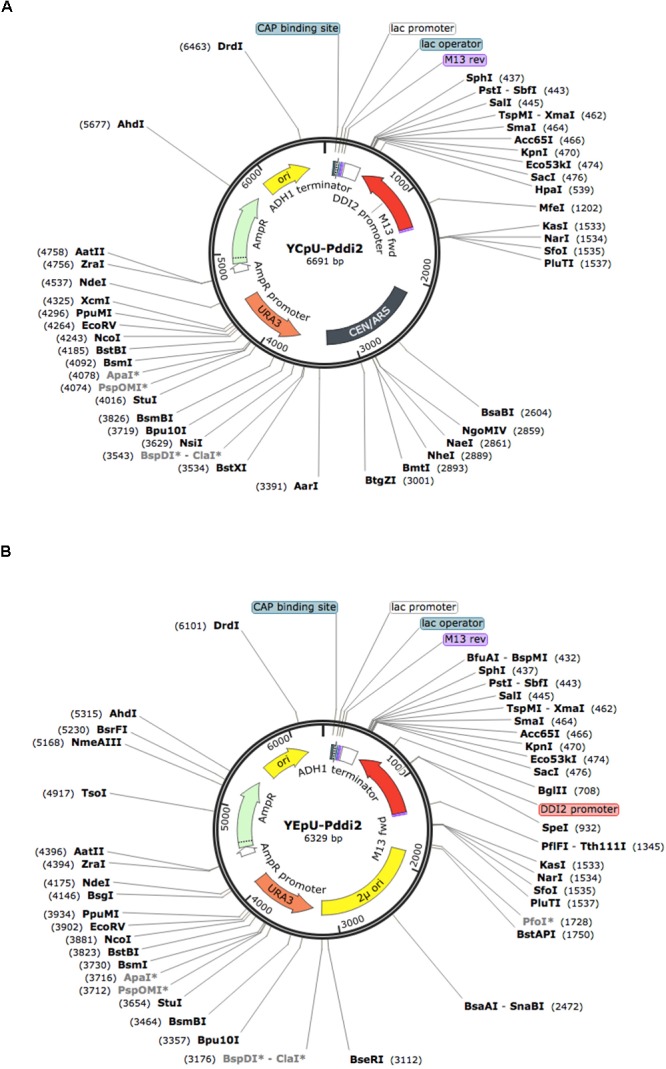
Physical maps of plasmids YCpU-P_DDI2_ and YEpU-P_DDI2_. **(A)** A single-copy plasmid YCpU-P_DDI2_. **(B)** A high-copy plasmid YEpU-P_DDI2._ Functional regions are marked in the inner circle and restriction enzyme recognition sites are marked. The maps were drawn with SnapGene (GSL Biotech LLC).

*Hin*dIII and *Eco*RI, two flanking restrictions sites in the MCS, were used to clone the *ADH1* terminator and the *DDI2* promoter (containing 888 nucleotides upstream of the *DDI2* translation start site), respectively. The entire inserts of resulting plasmids YCpU-P_DDI2_ (Figure 2A) and YEpU-P_DDI2_ (Figure 2B) were confirmed by sequencing (Supplementary Figure S1), which maintained most multiple cloning sites for future cloning of genes of interest.

### Validation of the Usefulness of YCpU-P_DDI2_ and YEpU-P_DDI2_

To assess plasmids YCpU-P_DDI2_ and YEpU-P_DDI2_, we cloned a *GFP* reporter gene into the above two plasmids to form YCpU-P_DDI2_-GFP and YEpU-P_DDI2_-GFP, respectively, which are used to monitor gene expression driven by the *DDI2* promoter in these vectors.

**FIGURE 3:**
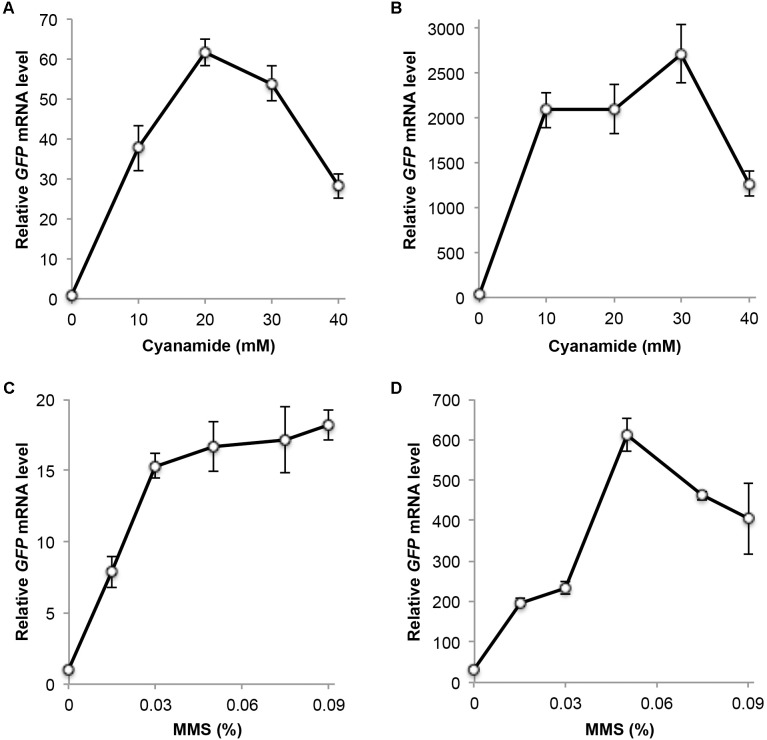
Relative *GFP* transcript levels measured by qRT-PCR. **(A)** Cells harboring plasmid YCpU-P_DDI2-_GFP in response to CY treatment. **(B)** Cells harboring plasmid YEpU-P_DDI2-_GFP in response to CY treatment. **(C)** Cells harboring plasmid YCpU-P_DDI2-_GFP in response to MMS treatment. **(D)** Cells harboring plasmid YEpU-P_DDI2-_GFP in response to MMS treatment. qRT-PCR was performed as described in Section “Materials and Methods” with *GFP*-specific primer pairs 5′-TCCGTTCAACTAGCAGACCA-3′ and 5′-GCCATGTGTAATCCCAGCAG-3′. The yeast *UBC6* mRNA was used as an internal control. The relative *GFP* mRNA level with untreated YCpU-P_DDI2_-GFP cells was set as 1. All data are an average of at least three independent experiments with standard deviations shown as error bars.

Based on previous studies, we chose CY or MMS treatment for 2 h as the optimal induction time. CY treatment at 20 mM induced *GFP* expression more than 60-fold in a single-copy plasmid (Figure 3A). In a multi-copy plasmid, the basal *GFP* level increased approximately 30-fold over the single-copy plasmid, and the CY treatment induced its expression by nearly another 100-fold (Figure 3B). A sublethal dose of 0.015% MMS induced *GFP* expression 7- to 8-fold in both single-copy (Figure 3C) and multi-copy (Figure 3D) plasmids, while 0.05% MMS induced *GFP* expression 17- to 20-fold in these plasmids (Figures 3C,D). It is noted that unlike endogenous *DDI2/3* expression, in which 0.06% MMS treatment reaches maximum level of induction (Figure 1A), the relative GFP mRNA level remains high by up to 0.09% MMS treatment (Figure 3C), when it causes severe cell death (Figure 1E). One possible explanation is that the *GFP* mRNA is more stable than *DDI2/3* mRNA. Indeed, GFP protein levels also remain high after lethal dose MMS treatment (see below).

**FIGURE 4:**
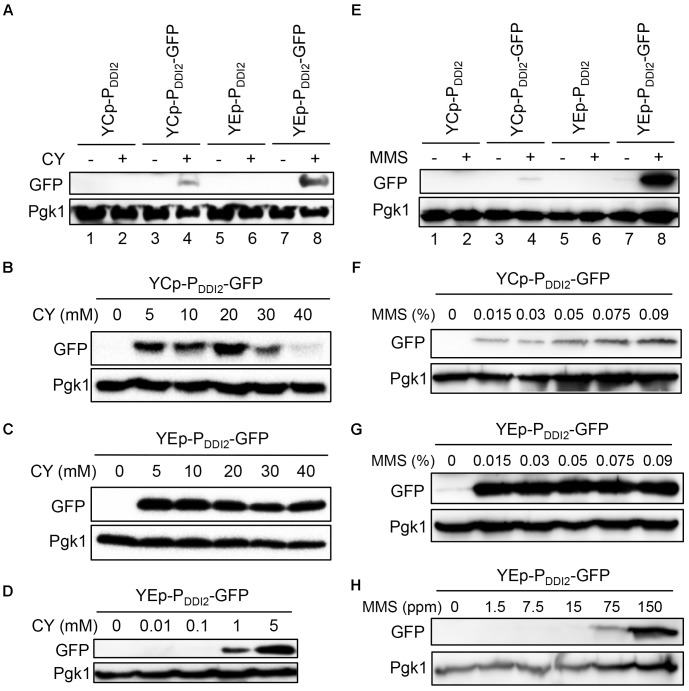
Western blot analysis of *P_DDI2_-GFP* gene products. **(A)** Cells harboring YCp- and YEp-based plasmids in response to 20 mM CY treatment. **(B)** Dose response of YCpU-P_DDI2-_GFP-transformed cells in response to CY. **(C)** Response of YEpU-P_DDI2-_GFP-transformed cells in response to high CY doses. **(D)** Response of YEpU-P_DDI2-_GFP-transformed cells in response to low CY doses. **(E)** Cells harboring YCp- and YEp-based plasmids in response to 0.015% MMS treatment. **(F)** Dose response of YCpU-P_DDI2-_GFP-transformed cells in response to MMS. **(G)** Response of YEpU-P_DDI2-_GFP-transformed cells in response to high MMS doses. **(H)** Response of YEpU-P_DDI2-_GFP-transformed cells in response to low MMS doses. The experimental protocol is described in Materials and Methods and all treatments were for 2 h. The anti-GFP monoclonal antibody B-2 was purchased from Santa Cruz (sc-9996) and the yeast anti-Pgk1 polyclonal antibody was a generous gift from Dr. W. Li (Institute of Zoology, Chinese Academy of Sciences, Beijing).

We also measured GFP protein levels under various induction conditions by western blot analysis. In cells harboring both single-copy and multi-copy plasmids, the GFP protein could not be detected under our experimental conditions without MMS or CY treatment. After 20 mM CY treatment for 2 h, the GFP protein was detected in both YCpU-P_DDI2_-GFP and YEpU-P_DDI2_-GFP, and the latter displays a higher GFP level (Figure 4A). For the single-copy-based P_DDI2_-GFP expression, GFP was detected after 5 mM CY treatment, and the maximum induction occurs at 20 mM CY (Figure 4B). Interestingly, for the multi-copy-based P_DDI2_-GFP expression, GFP was detected after CY treatment as low as 1 mM and the maximum induction occurred at 5 mM CY (Figures 4C,D). Similarly, 0.015% MMS could barely induce P_DDI2_-GFP expression in the single-copy plasmid and increased MMS concentrations further induced GFP levels (Figures 4E,F). In contrast, in the multi-copy plasmid 0.015% MMS induced the maximum level of P_DDI2_-GFP expression (Figure 4G), whereas the GFP protein could be detected after MMS treatment as low as 0.0075% (Figure 4H). These observations indicate that the target gene expression cloned in plasmids YCpU-P_DDI2_ and YEpU-P_DDI2_ could have rather different induction dynamics.

**FIGURE 5:**
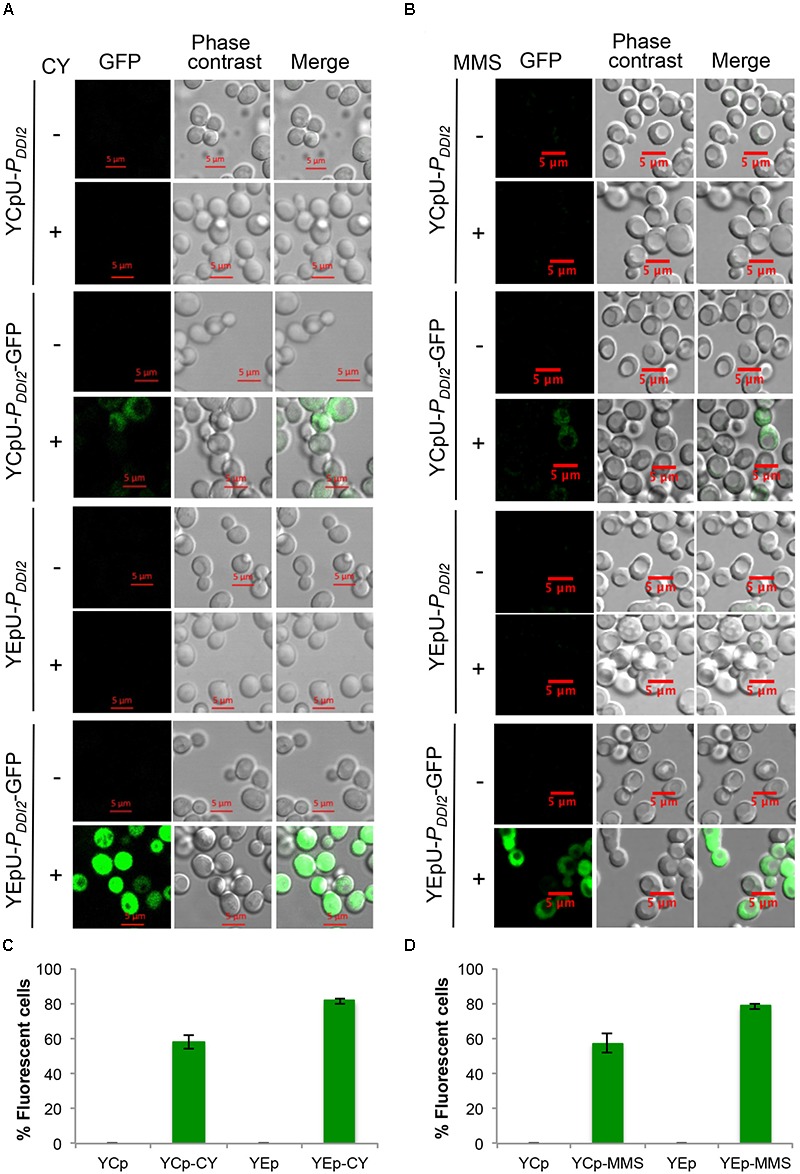
Fluorescent microscopic analysis of BY4741 transformants harboring *P_DDI2_-GFP* plasmids. **(A)** Representative images of cells with or without 20 mM CY treatment for 2 h. **(B)** Representative images of cells with or without 0.015% MMS treatment for 2 h. Plasmids are indicated on the left panel. **(C,D)** Quantitative analysis of percentage of fluorescent cells as shown in **(A,B)**, respectively. Cells transformed with empty vectors without the *GFP* gene are all negative for fluorescence and the data are not shown in the graphs.

Finally, we used fluorescence microscopy to monitor expression patterns of *GFP* cloned into single-copy and multi-copy plasmids driven by the *DDI2* promoter. This assay allows us to ask whether the target protein is produced at different levels in all cells or in different cell populations. It is seen from Figures 5A,B that none of the cells harboring the cloning vectors alone produced the GFP fluorescent signal, regardless of CY or MMS treatment. In the absence of CY or MMS treatment, cells harboring YCpU-P_DDI2_-GFP or YEpU-P_DDI2_-GFP plasmid also did not produce visible GFP signal. After 20 mM CY or 0.015% MMS treatment, approximately 40–60% cells harboring YCpU-P_DDI2_-GFP produced a relatively low GFP signal, whereas the majority of cells harboring YEpU-P_DDI2_-GFP produced a relatively strong GFP signal (Figures 5C,D). In addition, the CY treatment (Figure 5A) generated a much stronger signal than the MMS treatment (Figure 5B). The uneven expression levels among cells in a population may reflect their cell cycle stage or due to stochastic single-molecule events as reported in bacterial cells (Choi et al., 2008) that determine the individual cellular phenotype. Similar phenomena were also observed in our previous studies using different constitutive and inducible promoters (Tian et al., 2013).

### Replacing the Endogenous Yeast Promoter With a *DDI2* Promoter

**FIGURE 6:**
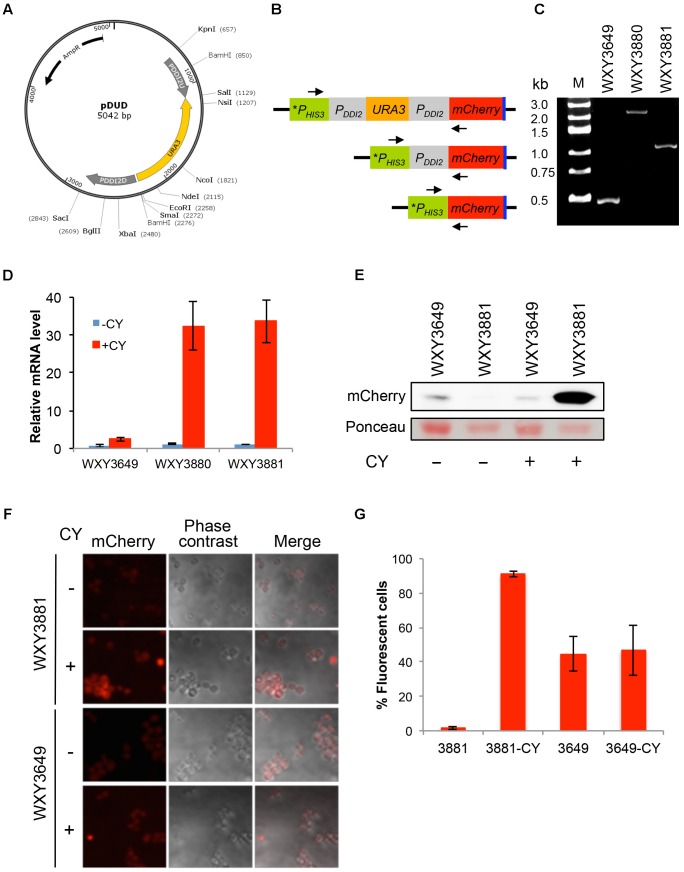
Construction and characterization of *DDI2*-based promoter shuffling. **(A)** The physical map of plasmid pDUD. The *P_DDI2_U-URA3-P_DDI2_D* cassette is shown in the inner circle. This cassette is used as a template to amplify a gene-specific cassette for yeast transformation. **(B)** Schematic diagram of pop-in and pop-out products at the *P_HIS3_-mCherry* locus in WXY3649. Arrows indicate forward and reverse primers 5′-TAGGAGTCACTGCCAGGTAT-3′ and 5′-TGCTTCACGTAGGCCTTGGAG-3′, respectively, used to perform genomic PCR to confirm the pop-in and pop-out products. **(C)** Agarose gel electrophoresis of genomic PCR products. Molecular size markers are indicated on the left. Predicted PCR product sizes are: WXY3649, 0.5 kb; WXY3880, 2.5 kb; WXY3881, 1.2 kb. **(D)** qRT-PCR analysis of *mCherry* expression in the pop-in (WXY3880) and pop-out (WXY3881) strains in response to 10 mM CY for 2 h. *mCherry*-specific primers are 5′-CAGACCGCCAAGCTGAAGGTGA-3′ and 5′-TCCCAGCCCATGGTCTTCTTCT-3′. The yeast *ACT1* mRNA was used as an internal control. The relative *mCherry* mRNA level with untreated WXY3649 cells was set as 1. The data are an average of three independent experiments with standard deviations shown as error bars. **(E)** Western blot analysis of mCherry-Myc levels in the pop-out strain in response to 10 mM CY treatment for 2 h using an anti-c-Myc monoclonal antibody 9E10 (Sigma, M4439). Ponceau stain was used prior to the western blot to serve as a loading control. **(F)** Representative images of cells with or without 5 mM CY treatment for 2 h. **(G)** Quantitative analysis of percentage of fluorescent cells as shown in **(F)**.

We previously developed a method to shuffle endogenous yeast promoters with a desired promoter to achieve optimal control of target gene expression without introducing additional genetic elements to the genome (Tian et al., 2013). In this study we wished to expand the repertoire by adding the *DDI2* promoter into the promoter-shuffling toolbox. To this end, plasmid pDUD was constructed by the same strategy as previously described (Tian et al., 2013), in which the *URA3* gene is flanked by two copies of the *DDI2* promoter (Figure 6A). Once integrated, the *URA3* gene along with one copy of the *DDI2* promoter can be popped-out through homologous recombination, leaving only one copy of the *DDI2* promoter to drive the target gene expression. To assess its usefulness, we amplified the *P_DDI2_U-URA3-P_DDI2_D* cassette by PCR, used it to transform strain WXY3649 and selected cassette integration at the *P_HIS3_-mCherry* locus. The resulting strain WXY3880 was then used to select 5-FOA-resistant derivatives like WXY3881. The anticipated genomic structures of each strain at the *P_HIS3_-mCherry* locus are illustrated in Figure 6B and were confirmed by genomic PCR (Figure 6C). qRT-PCR analysis of the *mCherry* expression revealed that the target gene was induced more than 30-fold in both pop-in (WXY3880) and pop-out (WXY3881) strains (Figure 6D). The experimental design also allowed us to compare the *DDI2* promoter with the *HIS3* promoter. As shown in Figure 6D, the basal levels of *mCherry* transcript driven by *HIS3* or *DDI2* are comparable. However, CY barely induced P*_HIS3_*-*mCherry* expression while *P_DDI2_-mCherry* expression was strongly induced. At the protein level, WXY3649 harboring *P_HIS3_-mCherry-Myc* produced detectable mCherry-Myc protein regardless of CY treatment. In sharp contrast, mCherry-Myc protein is undetectable in WXY3881 harboring *P_DDI2_-mCherry-Myc*, but it is massively accumulated after CY treatment (Figure 6E). At the individual cell level, untreated WXY3881 cells barely displayed fluorescent signal, while after CY treatment, fluorescent signals were detected in >90% cells (Figures 6F,G). It is interesting to note that under control of the *HIS3* promoter, approximately 50% cells showed fluorescent signals (Figure 6G) but their fluorescent intensity was much lower than CY-treated WXY3881 cells, regardless of CY treatment (Figure 6F). The above observations collectively indicate that one can replace desired endogenous promoters with the *DDI2* promoter so that the target gene expression is under strict control of CY.

## Discussion

In this study, we demonstrated that the *DDI2/3* gene can be induced not only by MMS by more than 100-fold as previously reported, but also by cyanamide by an astonishing 2000-fold and that this induction is very rapid upon treatment in cultured cells. In combination with the fact that cyanamide is not toxic within the entire range of optimal induction conditions and that it is very inexpensive, we envisage that the *DDI2* promoter-based expression system could be a useful experimental and industrial production tool. To facilitate utilization of this expression system by the yeast community, we developed both plasmid-based and chromosome-integration vectors and validated these systems by using fluorescent genes (*GFP* or *mCherry*) as reporters.

Several conclusions can be made from this study with regard to the plasmid-based *DDI2* promoter expression system. Firstly, the *DDI2* promoter cloned into a plasmid does not drive the reporter gene expression as high as it does in its native chromosome locus. We do not know the exact reason for the difference, as there are several variations between the two expression systems in addition to their “environment,” such as different terminators and target genes to be measured by qRT-PCR. Nevertheless, the induction by CY in both single-copy and multi-copy vectors is sufficient to strongly regulate the target gene to serve the purpose. Secondly, it is interesting to note that the basal-level expression from the multi-copy plasmid is about 30-fold higher than that from the single-copy plasmid, almost precisely reflecting the plasmid copy number difference (Futcher and Cox, 1984; Futcher, 1988), indicating that the target gene expression driven by the *DDI2* promoter is not saturated by the multiple gene copies. Furthermore, the induction after CY or MMS treatment is rather comparable between single-copy and multi-copy plasmids, further testifying that the expression is not saturated and perhaps still in a linear dose response range. Thirdly, analysis of the plasmid-based expression profiles reveals that single-copy and multi-copy vectors form a complementary system that could increase target gene expression by up to 2000-fold. Fourthly, in rare cases where CY cannot be used as an inducer, for instance to study biological effects of CY in budding yeast, sublethal MMS doses can still be used to increase target gene expression up to 200-fold, although it is unlikely to be applied to an industrial setting. Finally, at the optimal CY induction condition, almost all cells harboring YEpU-P_DDI2_-GFP produced a very strong fluorescent signal, suggesting that this inducible expression system can support very high industrial level production. Hence, if a study is to tightly control the target gene expression, we recommend the single-copy plasmid. On the other hand, if the purpose is to overexpress the target gene particularly in an industrial scale, we recommend the multi-copy plasmid.

Plasmid pDUD offers a complementary approach to studying yeast endogenous gene functions. By the promoter-shuffling method, one can replace the native gene promoter with the *DDI2* promoter so that the target gene is under strict control. Under normal growth conditions, the target gene would not be expected to produce sufficient product to carry out its function. Upon addition of a non-toxic dose of CY to the culture medium, the target gene expression is rapidly and massively induced.

## Author Contributions

AL and WX conceived and created the experimental design. AL, CZ, QW, WZ, ML, and MH conducted the experiments. AL and WX prepared the manuscript. WX supervised the project and provided funding and reagents.

## Conflict of Interest Statement

The authors declare that the research was conducted in the absence of any commercial or financial relationships that could be construed as a potential conflict of interest.
