# Supplementary material for: Utilization of a Strongly Inducible DDI2 Promoter to Control Gene Expression in Saccharomyces cerevisiae
Source: Front Microbiol. 2018 Nov 16;9:2736. doi: 10.3389/fmicb.2018.02736 (PMC6250804; doi:10.3389/fmicb.2018.02736)
Supplement: Supplementary file 1 [file Data_Sheet_1.PDF]

Supplemental Information

Figure S1. Insert DNA sequence information of plasmids YCpU-P_{DDI2} and YEpU-P_{DDI2}. (A) Physical map of the entire insert DNA. Red bar represents the *DDI2* promoter sequence from nt. -888 to -1 relative to the translation start site; blue bar represents the multiple clone site (MCS, nt. 890-946); and yellow bar represents the *ADH1* terminator region (nt. 947-1134). (B) Nucleotide sequences of the entire insert. Inserts from both YCpU-P_{DDI2} and YEpU-P_{DDI2} were sequenced and found to be identical.

A

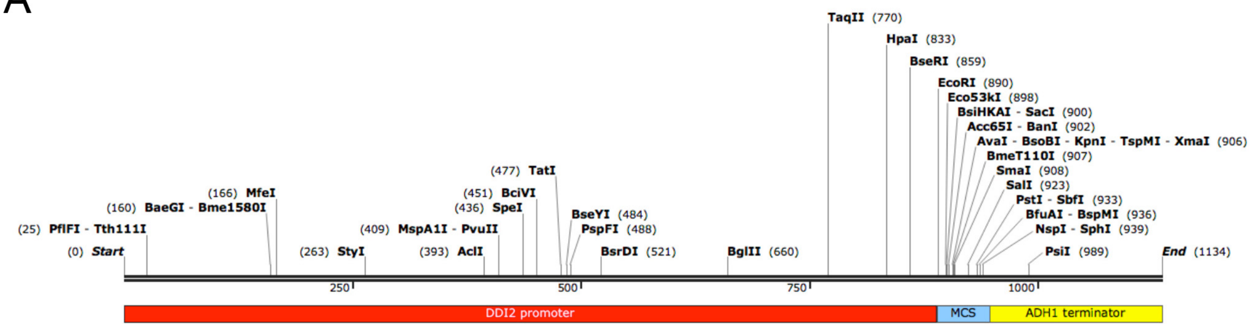

B

```
1    TTCAAAGGTT AAACTCGCTT AGACTATGTC TATAATATAA AAAAAAAATA GCTCTATTTC CGTTTCTTTT ATTCTATTTG ATATTTCCA

101  TCTTAATCAC GGATGTATAC TGATAATAGG GTTGACTGCG CCTGTACGGA TTACAGTGCC CTCTTCAATT GGAAAATCCA AGCTTTCAA

201  GTTATTCAAA GGATCCTCTA AGATAAAACA CAGATCGACA GATCCGAGAG TTGGCTTCTG TGCCTTGGGC TCAAATTCCT TTCCCACCT

301  ATTTTTCTGA CTCCAAAAAA AGACAGAGCC CTGCGATAGT TCCCGAATGT TGTAACATCA AAGCCAAGCA CTCCTTTATA GAAGTCGCA

401  TACTAGCAGC TGGTGAAACT ACAGGGTCTA AACTAACTAG TATCCATATC GTTTTGAGAG CATTGAAAGT ATACGGAGTA CAAGCTGGG

501  TTTTATCTTA ACAGCAATGA AAATCAACTT TCTAGACTGA ATCCCTCAAG AAAATTGCAA AAGACTAACC GATACTGGTT TAAAAGAGA

601  TATGCGGAGT TATACCATCA AACAACTTTG GACGGCCCCG AAACAAATGT CCGCAAAAAA GATCTTATTA AAGTGCATGG ACACTATCA

701  CAAAATACTC CACCGCACAA TAGTTTGTCG GGAAGTCATC AATCAATCTT GTACGAGCTT TACAAATAAC TTTTTAGGAT CGGTCCCCT

801  ATATAAATGG GTTAGTTTCC TTCTTCTTCT GTTAACATGA AGTTGCTTCG TACTGTTTTT TGCCTTGCTC CTCTTCAAAA GAATCAATC

901  CGGTACCCGG GGATCCTCTA GAGTCGACCT GCAGGCATGC AAGCTTGCGA ATTTCTTATG ATTTATGATT TTTATTATTA AATAAGTTA

1001 AGTGTATACA AATTTTAAAG TGACTCTTAG GTTTTAAAAC GAAAATTCTT ATTCTTGAGT AACTCTTTCC TGTAGGTCAG GTTGCTTTC

1101 ATGAGGTCGC TCTTATTGAC CACACCTCTA CCGG 1134
```

Figure S1
